# Ureteral fibroepithelial polyps with calculi: a case series

**DOI:** 10.1186/1752-1947-2-280

**Published:** 2008-08-26

**Authors:** Tahsin Turunc, Baris Kuzgunbay, Tuba Canpolat

**Affiliations:** 1 Department of Urology, Baskent University Faculty of Medicine, Ankara, Turkey; 2 Department of Pathology, Baskent University Faculty of Medicine, Ankara, Turkey

## Abstract

**Introduction:**

Fibroepithelial polyps of the ureter are benign tumors arising from the mesodermal tissue in the ureteral wall. Their etiology remains unknown. Hematuria and obstructive urinary symptoms are the most common findings. The treatment of choice is endoscopic resection, and the prognosis for patients with these lesions is excellent.

**Case presentation:**

We present three cases of fibroepithelial polyps associated with calculi in the distal part of the ureter. The patients were all women, aged 20, 45 and 52 years. Two patients were suffering from flank pain and dysuria while one patient was asymptomatic at the time of diagnosis. The patients were fully treated with endoscopic resection. To the best of our knowledge, this is the fourth report of adult ureteral fibroepithelial polyps associated with ureteral calculi in the English literature. The etiology, clinical features, diagnosis, and management of fibroepithelial polyps are discussed in this report.

**Conclusion:**

Whenever polypoid lesions are detected especially at the distal part of the ureter, benign fibroepithelial polyps should be kept in mind for differential diagnosis. Additionally, although rarely seen, the co-existence of ureteral calculi with fibroepithelial polyps should be borne in mind.

## Introduction

Fibroepithelial polyps (FEPs) are the most common benign lesions of the ureter. Most occur in the ureter and renal pelvis in adult patients, while a few occur in the posterior urethra or bladder, generally in children [1]. However, FEPs of the ureter accompanied by calculi are rare. We review our experiences with three patients having FEP associated with calculi in the distal ureter to define this entity more clearly and its outcome following treatment.

## Case presentation

### Case 1

**Figure 1:**
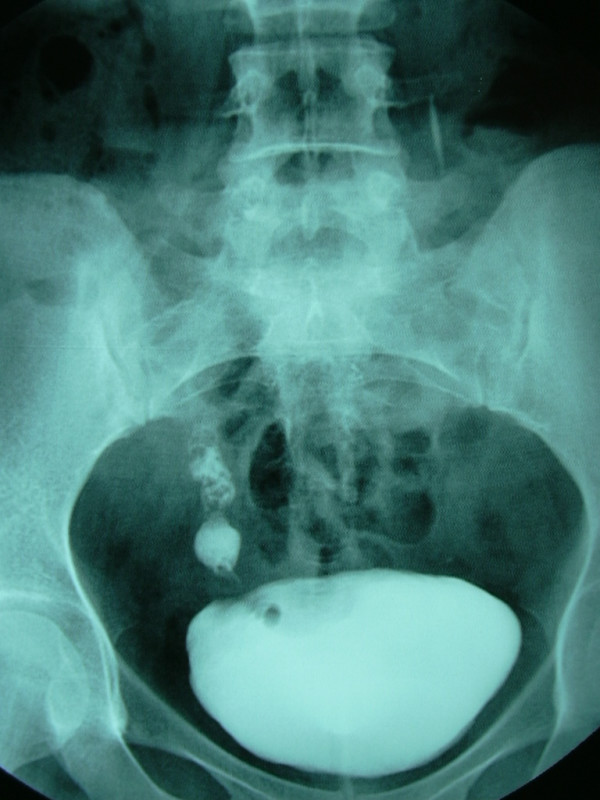
**Intravenous urographic demonstration of fibroepithelial polyps in distal ureter with filling defects**.

**Figure 2:**
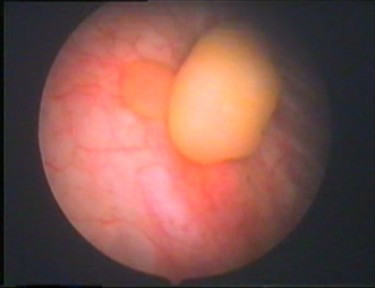
**Cystoscopic visualization of fibroepithelial polyps prolapsing into the bladder from the right ureteral orifice**.

A 20-year-old woman presented to our clinic with intermittent right flank pain and dysuria. She had undergone an extracorporeal shockwave lithotripsy for a kidney stone 5 months before the current admission. Urine analysis showed mild microscopic hematuria. Intravenous urography demonstrated a 12-mm calculus in the right distal ureter, focal ureteral dilatation in the proximal part of the ureter, smoothly marginated tubular filling defects, and mild hydronephrosis. Also, there was another irregular filling defect at the level of the right ureteral orifice (Fig. 1). On cystoscopic examination of her bladder, there were two polypoid masses, grayish-white in color, about 2 cm long and 0.5 cm wide, prolapsing into the bladder from the right ureteral orifice with a thin stalk (Fig. 2). A urine sample was taken for immediate cytology and the polyps were grasped with forceps for traction and resected over the root area through the ureteroscope and manipulated for frozen biopsy. The results of the urine cytology were negative, and the analysis of the frozen section demonstrated a benign FEP. Then the ureteroscope was inserted into the right ureter through the guide wire and the calculus was fragmented by pneumatic lithotripsy and fragments were extracted with a basket. About 1 cm proximally to the calculi, multiple millimetric polypoid structures were exposed and all of them were resected as described above. The bases of the polyps were coagulated with an electrode to stop bleeding and prevent recurrences. A ureteral stent was placed to provide urine drainage and to avoid probable obstruction caused by edema, then removed 24 hours later.

The final pathology report showed FEP. Histologically, the polyps had a core of loose fibrovascular stroma covered by a layer of normal transitional uroepithelia, associated with edema, congestion, and mononuclear cell infiltration. No significant cellular atypia or cytologic abnormality was observed.

### Case 2

A 45-year-old woman presented to our clinic with left flank pain, dysuria, and hematuria. *Escherichia coli *was detected in a urine culture and treated with levofloxacin (500 mg PO q.d.) for 2 weeks. An intravenous urogram (IVU) showed two 0.6 cm calculi in the left distal ureter, a tubular filling defect proximal to the calculi, and moderate hydronephrosis.

**Figure 3:**
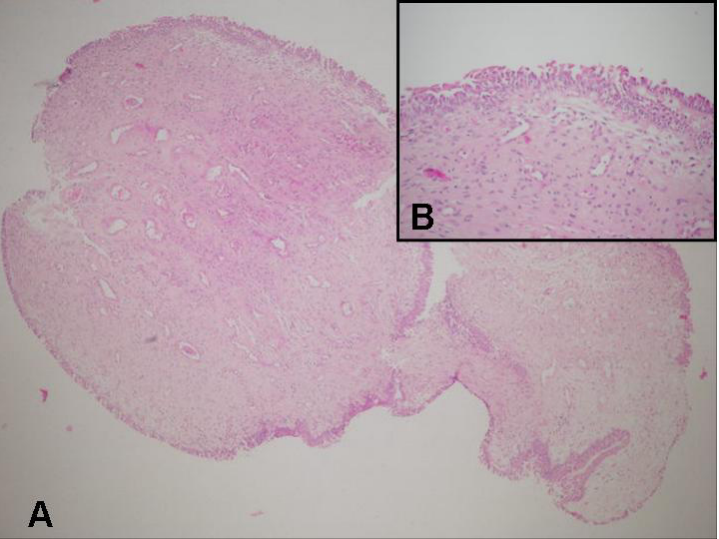
Fibroepithelial polyp of ureter that has a loose fibrovascular stroma (A) (hematoxylin and eosin, ×40) and covered by transitional epithelium (B) (hematoxylin and eosin, ×200).

A left ureteroscopy showed a polypoid mass, grayish in color, about 1 cm long and 0.3 cm wide, with a thin stalk that projected into the lumen in the distal ureter. A urine sample was obtained for cytology, and then the polyp was grasped with forceps for traction and resected over the root area through the ureteroscope and manipulated for frozen biopsy. Urine cytology was negative and frozen biopsy results were in favor of benign FEP. The base of the polyp was coagulated by an electrode to stop bleeding and prevent recurrences. Then, pneumatic lithotripsy was performed, the resultant fragments were extracted, and double J stents were inserted for 4 weeks. The final pathology report revealed a FEP (Fig. 3).

### Case 3

A 52-year-old woman presented to our clinic with an asymptomatic left distal ureteral stone diagnosed incidentally. IVU showed a calculus 12 mm long and located in her left distal ureter without any ipsilateral urinary drainage, indicating no renal dysfunction and right renal calculi. On cystoscopic examination, 2 polyps were detected, 5 mm in diameter and prolapsing into the bladder from the left ureteral orifices. The polyps were resected in the same way as described above and the calculus was managed with ureteroscopic lithotripsy. The final pathology report revealed a FEP.

IVU was performed in all three patients 2 months after the operation; no pathological finding was detected except focal dilation in the distal ureter in Case 1 and residual dilatation in the ureter and the kidney in Cases 2 and 3.

All patients subsequently underwent annual radiographic follow-ups with IVU, and no recurrences were detected.

## Conclusion

Ureteral FEP was first reported in 1932 [2], and since then, there have been approximately 236 scientific papers on fibroepithelial polyps. Fibroepithelial polyps are rare, benign, mesodermal tumors of the urinary tract that are histologically composed of fibrous stroma covered with a transitional urothelium. They are considered the most common benign lesions of the ureter among other benign lesions such as leiomyomas, lymphangiomas, and neurofibromas. They are often smoothly marginated and cylindrical, sessile, or even frond like [1]. Because of their histologic organization, FEPs are classified as benign hamartomas; however, malignant degeneration and cystic transformation have also been reported [3,4].

Fibroepithelial polyps commonly present in adults in the third to fifth decades with a male-to-female ratio of 3:2. In adults, most FEPs occur in the ureter; 62% of these polyps are located in the upper ureter or uretero-pelvic junction, 15% are in the renal pelvis, and a small percentage is in the bladder or posterior urethra [1]. Fibroepithelial polyps of the lower urinary tract usually occur in the posterior urethra, most often in children. They usually appear as solitary polyps; however, rare cases of multiple and bilateral appearances have been reported [3,5].

Although the etiology of FEPs is unclear, they are thought to be either congenital slow-growing lesions or lesions that develop as a result of chronic urothelial irritants, such as infection, inflammation, calculi, or obstruction. The most significant signs and symptoms of the polyps are hematuria and flank pain [1]. The pain is characteristically intermittent and colicky due to partial obstruction. Urinary frequency, dysuria, and pyuria are other less common findings [1].

On IVU or retrograde urograms, FEPs appear to be long, smooth ureteral filling defects; their position may change between images; and they are associated with varying degrees of hydronephrosis [6]. It is important to distinguish FEPs from upper urinary tract carcinomas because management and prognosis can be significantly different. Debruyne and associates reported that unnecessary nephroureterectomies were performed in 42 of 112 patients (37%) with FEP because of an uncertain pre-operative diagnosis [7].

In the past, management of FEP was excision of the polyp and reanastomosis with an open procedure. Recently, with the advent of ureteroscopes, minimally invasive endoscopic treatment has become more popular. Usually, the polyps are grasped with forceps for traction and resected over the root area through the ureteroscope, and the base is fulgurated to prevent recurrence. The holmium:YAG laser is another modality for endoscopic resection. Carey and Bird successfully ablated multiple polyps in one ureter by using the holmium laser, and removed each polyp from the ureteral wall with grasping forceps. Also, ureteral stones are removed concurrently with a basket and a ureteral access sheath is used to facilitate the multiple passes of the ureteroscope and the removal of the polyps and stones from the proximal ureter [8]. Percutaneous antegrade excision should be available for treating polyps in the renal pelvis and the upper ureter [3]. Laparoscopic surgery might be preferred over open surgery when the polyps are too large to be fully cleaned by endoscopic surgery.

Although close follow-up was recommended in the literature because of the risk of recurrence, the duration and frequency of follow-up are not clear. Although some studies have suggested cytological evaluation of urine in the postoperative follow-up, we do not agree with this because of the benign nature of FEP. Some studies have suggested control ureteroscopy associated with IVU in the follow-up [8]. We think that yearly IVU should be helpful in the follow-up after the initial IVU 2 to 3 months after the endoscopic resection of FEP. However, the follow-up period might be changed depending on the clinical progression, signs and symptoms of the disease. Interestingly, although fibroepithelial polyps were solitary in children, in the cases presented here they were multiple, located in the distal part of the ureter and associated with an adjacent ureteral calculus; however, this scenario has rarely been reported in the literature. There have only been three prior case reports of ureteral FEP with adjacent urolithiasis in adults [[8,9], 10], and long-term or repeated inflammation of ureteral tissue by urinary crystals, calculi, stents, and infections has been implicated.

Since chronic irritation has been reported to be an etiological factor in fibroepithelial polyps, calculi in the ureter may be responsible for the formation of FEP; however, the opposite might also be true. In the cases presented here, urinary retention and chronic irritation due to the obstruction formed by the ureteral stones might have been responsible for the ureteral polyps, but the accepted theory in the literature is stone formation due to urinary retention caused by FEP. In both situations, endoscopic management of FEP and calculi is safe, effective, and minimally invasive. We reviewed our experience in cases of FEP associated with calculi in the distal ureter and attempted to define this entity and its outcome more clearly after treatment. When a calculus and an accompanying polypoid mass are detected in the ureter, FEP, which is histologically benign in nature, should be kept in mind in differential diagnosis and both polyps and calculi should be managed at the same time, if possible.

## Abbreviations

FEP: Fibroepithelial polyp; IVU: Intravenous urography.

## Competing interests

The authors declare that they have no competing interests.

## Authors' contributions

TT reviewed the literature, conceived and drafted the manuscript. BK and TC examined the patient, helped to record the data and prepare the manuscript. All of the authors read and approved the final manuscript.

## Consent

Written informed consent was obtained from the patients for publication of this case series and accompanying images. A copy of the written consent is available for review by the Editor-in-Chief of this journal.
